# Moral Injury and Post‐Traumatic Stress Disorder in War: The Effect of Marital Status and Previous Genocidal Trauma

**DOI:** 10.1002/ijop.70204

**Published:** 2026-03-20

**Authors:** Larysa Zasiekina, Serhii Zasiekin, Victor Kuperman

**Affiliations:** ^1^ University of Exeter Exeter UK; ^2^ Lesya Ukrainka Volyn National University Lutsk Ukraine; ^3^ University College London London UK; ^4^ McMaster University Hamilton Canada

**Keywords:** genocidal trauma, intergenerational transfer, moral injury, PTSD, the Holodomor

## Abstract

This study examines the intergenerational transfer of the genocidal trauma of the Holodomor (1932–33) and explores how marital status moderates its impact on moral injury and post‐traumatic stress disorder (PTSD) in the context of the ongoing Russia‐Ukraine war. Moral injury, distinct from PTSD, arises from the violation of moral beliefs, leading to emotional distress characterised by guilt, shame, anger, disgust and a sense of betrayal. While previous research predominantly focused on direct survivors of genocide, this study expands the understanding of moral injury to their descendants, particularly the third and fourth generations, highlighting the often‐overlooked familial dynamics involved. Through a sample of 1857 participants, our findings reveal that married descendants of Holodomor survivors exhibit significantly higher levels of moral injury when familial genocidal trauma is present, contrasting with non‐married individuals who show no significant difference. This suggests that marital status plays a vital role in shaping the emotional burden of inherited moral injury, as these individuals grapple with the dual responsibilities of familial protection and the distress of genocidal trauma. Our results indicate that the interaction of genocidal trauma and marital status does not extend to PTSD. These findings emphasise the need for targeted family‐based interventions to address the complexities of intergenerational moral injury.

## 1 Introduction

### 1.1 Moral Injury and Post‐Traumatic Stress Disorder in War and Genocide Settings

Moral injury is an emotional distress arising from directly experiencing or witnessing others' behaviour violating an individual's moral principles and values. Research underscores that moral injury is a potential dimensional clinical problem that impairs individual functioning and manifests a loss of faith in personal or collective rights (Litz and Kerig 2019). Moral injury results in poor health outcomes, including depression, substance use and suicidality (Hall et al. 2022). Recently, moral injury has gained increasing formal recognition in both research and clinical practice. Although not classified as a psychiatric disorder, it is acknowledged as a significant psychological and ethical stressor that can profoundly impact emotional well‐being, social functioning and moral reasoning. The DSM‐5‐TR Z65.8 code ‘Moral, Religious or Spiritual Problem’, emphasises that moral injury arises from violations of deeply held ethical or moral values rather than being a traditional mental disorder (Mattson et al. 2025).

Evidence suggests that moral injury occurs after experiencing traumatic situations where external circumstances force an individual's behaviour. This is the reason why posttraumatic stress and moral injury are defined as different but still co‐occurrence concepts (Molendijk 2022). Despite the strong association between moral injury and posttraumatic stress disorder (PTSD), recent findings suggest that moral injury can be conceptualised as a unique form of emotional distress, distinguishable from PTSD and primarily characterised by feelings of guilt, shame, anger, disgust and a sense of betrayal. The key differences between PTSD and moral injury can be delineated based on their conceptual boundaries, symptomatology, measurement, neural underpinnings and treatment (Zasiekina, Kokun, et al. 2023; Zasiekina, Zasiekin, and Kuperman 2023).

Traumatic situations that involve individuals in morally undesirable behaviour often occur during wartime. In a recent paper, Fleming (2021) referred to absurd circumstances (e.g., clashes of values, competing moral expectations and moral paradoxes) as a precondition for moral injury, expanding the concept of potentially morally injurious events (e.g., perpetrating harm; failing to prevent harm; witnessing atrocities; feeling of being abandoned or betrayed) fully described by Litz et al. (2009). By broadening the definition to include these moral paradoxes, Fleming (2021) highlights that moral injury can arise from explicit moral transgressions and moral dilemmas where the morally correct decision is impossible because the context of war itself is inherently unjust. Furthermore, Fleming (2022) describes the potential disruption of core moral beliefs about oneself and others in veterans, which can shake an individual's moral compass, ultimately leading to the development of complex moral injury.

Although most literature highlights the clinical cases of moral injury in the military population, recent studies show that it can occur in the civilian population across the age range exposed to continuous traumatic stress during ongoing violence, wars and genocides (Fani et al. 2021; Zasiekina, Kokun, et al. 2023; Zasiekina, Zasiekin, and Kuperman 2023). The findings from our previous qualitative and quantitative studies of moral injury show the expression of anger, contempt, disgust, decreased empathy and embarrassment in genocide survivors of the Holodomor and the Holocaust (Zasiekina and Zasiekin 2020, Zasiekina et al. 2021). Exposure to continuous traumatic stress might lead to an accumulative effect of chronic moral injury that can emerge as continuous moral injury in war and genocide settings (Leshem et al. 2025; Zasiekina et al. 2024).

### 1.2 Intergenerational Transfer of Holodomor Genocidal Trauma in Families

Genocidal trauma arises from the definition of genocide as set out in the Genocide Convention, which defines genocide as acts committed with the intent to destroy, in whole or in part, a national, ethnic, racial, or religious group. Accordingly, genocidal trauma refers to the psychological and emotional aftermath of exposure to actions intended to destroy such a group (Hamburger 2017). This trauma not only impacts direct victims but also profoundly affects their descendants, who often inherit post‐traumatic symptoms and moral injuries tied to these atrocities (Danieli 2009; Maercker 2023; Starrs and Békés 2025).

Prior research on the intergenerational transmission of genocidal trauma has focused primarily on the first and second generations, with far less attention given to the third and fourth generations. Studies on the third generation are limited and inconsistent (Freud and Berant 2023; Scharf 2007), and research on the fourth generation is even scarcer. Haladjian (2020) examined Armenian‐American participants aged 17–75 with relatives who survived the Armenian Genocide, investigating secondary trauma symptoms across second, third and fourth generations. While family silence regarding the genocide was not directly associated with secondary trauma, bicultural identity emerged as a potential protective factor, whereas higher Anglo‐acculturation was linked with increased secondary trauma in later generations. These findings suggest that acculturation may have differential effects on mental health depending on cultural context, pre‐migration trauma exposure and post‐migration stressors such as limited social support and perceived discrimination. Similarly, Shrira et al. (2024) found that ancestral PTSD among Holocaust survivors contributed to latent vulnerability in offspring three generations later. Intergenerational trauma has also been observed in refugee and displaced families, although research in these populations remains limited, particularly studies including children and youth and examining both risk and protective factors shaping mental health outcomes (Sangalang and Vang 2017). Some evidence further suggests that higher educational attainment may serve as a protective factor in the intergenerational transmission of trauma, whereas female gender may be associated with increased risk of adverse mental health outcomes (Chen et al. 2008; Zasiekina et al. 2021). Collectively, these studies underscore the potential relevance of intergenerational genocidal trauma that may amplify predisposition to mental health difficulties in descendants.

Multiple biopsychosocial frameworks attempt to explain how genocidal trauma passes from one generation to the next, highlighting its complexity. Psychodynamic theories propose that unresolved trauma disrupts family functioning, fosters silence around painful events and transmits unconscious fears to children (Danieli 1998; Volkan 2001). Developmental and attachment perspectives similarly emphasise family mechanisms, suggesting that trauma undermines caregiving, damages secure attachment and increases emotional dysregulation, thereby heightening children's mental health risks (Bifulco 2021; Dekel and Goldblatt 2008).

Biological research, particularly in epigenetics and neurobiology, demonstrates that genocidal trauma can produce enduring gene expression changes through processes like DNA methylation and make effects on brain structure, connectivity and stress reactivity. These alterations may be inherited by subsequent generations and influence stress responses, immune functioning and metabolic and neurodevelopmental pathways (Addissouky et al. 2025; Bolouki 2023; Cao‐Lei et al. 2022; Yehuda et al. 2014).

Sociocultural influences, such as collective memories, cultural narratives and social stigma, also contribute to trauma transmission (Danieli 1998; Hirsch 2012; Volkan 2001). In communities affected by genocide, these shared narratives often shape cultural identity and influence the psychological well‐being of descendants (Gorbunova and Klymchuk 2020; Danieli et al. 2016; Hirsch 2012; Yehuda and Lehrner 2018; Zasiekina and Zasiekin 2020).

Despite these biopsychosocial advances, relatively little research has examined how family dynamics specifically contribute to the intergenerational transmission of moral injury. Moreover, much of the literature addresses both collective and individual suffering, while often neglecting the familial dimensions of genocidal trauma (Newman and Erber 2002). To take a recent example, Maercker (2023) discusses the psychological impacts of fear and distrust in family settings during political oppression in Eastern Europe. However, the particular role of family has not been discussed. This oversight in recent literature is significant because family environments are central to how trauma and moral injuries are passed down, influencing emotional support systems, coping mechanisms and vulnerability to further trauma (Fitzgerald et al. 2020). Within this context, marital relationships may represent a particularly salient setting in which inherited trauma is interpreted. Being married may intensify feelings of moral responsibility and obligation toward family continuity, potentially amplifying the moral burden associated with ancestral suffering even when PTSD symptoms are not elevated. Marital status has been recognised as a factor influencing family emotional dynamics and trauma‐related mental health symptoms, with evidence suggesting that married individuals may have higher odds of PTSD compared to divorced individuals (Watkins et al. 2017; Sh Abukar et al. 2025); however, its role in moral injury remains insufficiently examined.

Marital status can influence an individual's experience of moral injury, particularly when family members are exposed to violence, oppression, or death. The marital relationship provides emotional support, yet can also increase pressure, particularly in the offspring of genocide survivors, where the responsibility to protect loved ones may amplify feelings of guilt and helplessness (Mooren et al. 2022). Married individuals might feel an intensified moral burden to protect their family, leading to greater vulnerability to moral injury, particularly when confronted with situations that challenge their moral beliefs or capacity to act. Exploring marital status as a risk factor for moral injury can inform targeted interventions that mitigate these effects, improving the mental health and resilience of those affected by intergenerational trauma.

This study proposes to examine how the genocidal trauma of the Holodomor (1932–33), the Great Famine, continues to affect descendants of Ukrainian survivors, focusing on the role of marital status in shaping the transfer of moral injury. The Holodomor, a man‐made famine orchestrated by the Soviet regime, devastated Ukraine, resulting in the deaths of millions and collective psychotrauma across generations (Kis 2021; Naimark 2015).

The experience of forced displacement and separation from loved ones during the Holodomor mirrors the current displacement crisis in Ukraine, where millions are again fleeing due to the Russia‐Ukraine war. This research seeks to understand how the Holodomor's legacy interacts with marital status to affect aggregating moral injury in descendants in the context of their exposure to multiple war‐related traumas. By examining these interactions, we hope to illuminate the complex ways in which familial dynamics, particularly marital relationships, shape the transfer of moral injury across generations. Specifically, the research examines if there is an interaction effect between the genocidal trauma of the Holodomor and marital status on moral injury and PTSD and if current war‐related events can re‐activate them.

## 2 Methods

This study is based on the description of methods and an extension of the dataset reported in Zasiekina, Kokun, et al. (2023) and Zasiekina, Zasiekin, and Kuperman (2023). The current study differs from previous research using the same dataset by focusing on the intergenerational pathways of moral injury and PTSD rather than on current war‐related moral injury and PTSD and by exploring the moderating effect of marital status.

The study was reviewed and approved by the Research Ethics Committee (#03‐24/04/1070) at Lesya Ukrainka Volyn National University, Ukraine and the Research Ethics Board of McMaster University, Canada (#6045).

### 2.1 Participants

As of 03 March 2023, a total of 2157 participants contributed to the Narratives of War project. All of them completed a narrative writing task, and 2009 of them also submitted demographic and psychological questionnaire data; see descriptions of tasks below. The respondents ranged in age from 12 to 86 years. We removed one participant with an invalid age value and 151 participants younger than 18. The analyses below are based on the resulting pool of 1857 participants who completed all tasks. Table 1 summarises descriptive statistics of the demographic and questionnaire data.

**TABLE 1:** Descriptive statistics of dependent variables and predictors in the study (*N* = 1857).

Predictor	Levels	*N* per level	Min, median, mean (SD), max
Gender	Female	1524	
	Male	332	
	Other	1	
Age		1857	18, 32, 33 (11), 86
Education	Secondary	344	
	College	557	
	Bachelor	302	
	Master, specialist, PhD	654	
Marital status	Married	941	
	Not married (single, divorced, widowed)	916	
Displaced	No	1028	
	Yes	829	
Occupation status*	Occupied	960	
	Deoccupied	308	
	Never occupied	589	
Region	Central	312	
	Eastern	355	
	Northern	294	
	Southern	669	
	Western	227	
Family trauma	None	1114	
	Holodomor	276	
	Other	467	
Dependent variable			
PCL‐5 score		1857	0, 45, 44.13 (16.32), 80
MISS score		1857	10, 43, 43.33 (13.17), 92

* Occupation status is determined per oblast at the response submission date. Subsample sizes for categorical variables are given per level.

### 2.2 Procedure

This online study was administered in May 2022–August 2024. Participants were recruited by the Ukrainian Psychotrauma Center via its website and Facebook page and were provided with an information sheet and a consent form. They communicated their consent by filling the respective field in the online Google form. Upon consent, participants were invited to fill in their demographic data and questionnaires through Google Forms. They were also asked to complete the narrative writing task, that is, describe their experience of the Russia‐Ukraine war in at least 300 words. Participants were paid UAH200 as compensation for their time.

### 2.3 Materials and Variables

Two psychological questionnaires were used in the study. One is the standard PTSD Checklist for DSM‐5 (Weathers et al. 2013), labelled here as PCL‐5. The questionnaire consists of 20 Likert‐scale questions asking how much the respondent has been bothered by a given problem in the past month, from 0 (‘not at all’) to 4 (‘extremely’). The PCL‐5 total scores range from 0 to 80, with a higher score corresponding to a higher total severity of PTSD symptoms. The reliability analysis of PCL‐5 scores based on the subset of the present data pool (Zasiekina, Kokun, et al. 2023; Zasiekina, Zasiekin, and Kuperman 2023) revealed very high reliability (ICC(2, *k*) = 0.92, 95% CI [0.91, 0.92]) in line with the earlier report by Shevlin et al. (2018).

The second questionnaire is the Military Version Short Form of the Moral Injury Symptom Scale (Koenig et al. 2018), labelled here as MISS. This Likert scale consists of 10 statements (e.g., ‘I feel betrayed by leaders who I once trusted’) with response options ranging from 0 (‘strongly disagree’) to 10 (‘strongly agree’). The total score ranges from 10 to 100, with higher values corresponding to a higher level of moral injury. The Ukrainian version of the MISS‐M‐SF scale demonstrates sound psychometric properties (Zasiekina et al. 2024) and was adopted to assess moral injury in military and civilian populations according to the procedure suggested by Fani et al. (2021). The reliability of the MISS scores in the subset of the present data examined in Zasiekina, Kokun, et al. (2023) and Zasiekina, Zasiekin, and Kuperman (2023) was moderate (ICC(2, *k*) = 0.53, 95% CI [0.47, 0.59]).

#### 2.3.1 Dependent Variables

PCL‐5 and MISS test scores were dependent variables in this study.

#### 2.3.2 Independent Variables

Our primary interest is in the interaction of the variable representing the genocidal trauma in the family history and marital status of the respondent as a predictor of the severity of moral injury and PTSD symptoms. One critical independent variable was the presence of genocidal trauma in the family history of the respondents. Participants provided free‐form responses to this question, which were later processed and tabulated by research assistants (see Table 1 for the list of outcomes). Another critical variable was the marital status of the respondent (aggregated options Married and Not Married). To estimate the predictive power of the interaction, we accounted for a range of risk factors among civilians experiencing continuous traumatic stress.

Previous research (Zasiekina, Kokun, et al. 2023; Zasiekina, Zasiekin, and Kuperman 2023) enabled us to focus on selected geographic and demographic factors as independent variables of potential influence. These included Gender (with male, female and other as options), age and Education (with secondary, college/vocational secondary, bachelor's degree and higher university degree as levels). To link participants to various circumstances of the ongoing war, we further identified the respondent's reported pre‐war place of residence against five major geographical regions of Ukraine. We further annotated the occupation status of the specific administrative unit (oblast) at the time of the respondent's study completion (Occupied by the Russian troops, Deoccupied, Never occupied). Finally, we asked whether a respondent was displaced (internal displacement or emigration) when responding. See Table 1 for descriptive statistics of each variable.

#### 2.3.3 Statistical Considerations

We used multiple linear regression to estimate the interaction effect of genocidal trauma and marital status on individual scores in two psychological questionnaires while accounting for the effects of other predictors. All analyses were conducted in the statistical software environment R v 4.4.1 (R Core Team 2024). Because of the relatively large number of predictors, we approached regression modelling in two steps. The first step fitted the regression model to one of the scores using the full set of predictors described above. Then, the backward elimination algorithm was applied to retain only those predictors that decrease the model performance if deleted. The algorithm uses the Akaike Information Criterion (AIC) as a goodness‐of‐fit metric and is implemented in function step AIC of the MASS library v 7.3–60 (Ripley and Venables 2022). The second step of the procedure was to fit the linear regression model only with those predictors that were retained in the first step. The outcomes of these models are reported below. For categorical predictors, we report effects both in the original test scale and standardised, in units of standard deviation. The standardised coefficients also serve as estimates of the effect size.

The power analysis of linear regression indicates that the present sample, *N* = 1390, enables the detection of a very small effect in a linear regression model (*f* ^2^ = 0.01) with the conventional 80% statistical power and the 5% significance level: This effect size is 20 times smaller than what Cohen (1992) considers a small effect. We conclude that this study is properly powered.

## 3 Results

The distribution of responses to the critical variable genocidal trauma is reported in Table 1. The majority (1114 out of 1857) did not report any traumatic event in their family history, 276 indicated Holodomor and 467 provided responses that we classified as Other. These responses included indication of the Holocaust (20 responses, or 1% of the total participants), the current events of the Russia‐Ukraine war, which do not qualify as genocidal trauma, or non‐valid responses. Below, we only consider data from the 1390 participants who responded None or Holodomor: other responses were either too infrequent for the statistical analyses or did not fall under our definition of past genocidal trauma of the Holodomor.

### 3.1 Severity of PTSD Symptoms

The backward elimination algorithm of the linear regression model fitted to PCL‐5 scores retained six out of eight predictors based on the changes in the AIC score: Age (linear and quadratic terms), Gender, displacement status, Education, occupation status and importantly, genocidal trauma. The second regression model only used these six predictors of PCL‐5 scores.

The effects of the control predictors replicated the earlier findings of Zasiekina, Kokun, et al. (2023) and Zasiekina, Zasiekin, and Kuperman (2023). The severity of PTSD symptoms, as measured by PCL‐5 scores, showed a non‐linear increase with age (a more significant increase from young to mature adulthood and a plateau from mature to senior adulthood). Severity was higher for women than men, displaced rather than non‐displaced individuals, individuals with lower education levels and individuals living in the occupied territories at the time of writing, see Table 2. The key finding is that individuals who reported no genocidal trauma showed numerically lower PCL‐5 scores compared to those who indicated Holodomor (*b* = −1.833, SE = 1.090, *t* = −1.681, *p* = 0.093). The effect was small and amounted roughly to 2 points on the 0–80 point scale of PCL‐5; the standardised effect of the genocidal trauma index amounted to only 0.05 SD of the dependent variable. Also, the effect did not reach the 5% statistical significance threshold. Furthermore, the interaction between family trauma and marital status was not a significant predictor of the PCL‐5 scores (*β* = 2.90, SE = 2.17, *t* = 1.4, *p* = 0.182), and neither were interactions between family trauma and displacement status or education (models not shown). We conclude that the presence of genocidal trauma trends in the expected direction but does not substantially contribute to PTSD symptoms in the Ukrainian civilians.

**TABLE 2:** Coefficients of the regression model fitted to PCL‐5 scores.

Predictor	*b*	SE	*β*	*t*	*p*
(Intercept)	30.398	4.499	—	6.757	< 0.001
Age (linear)	0.638	0.219	0.12	2.913	0.004
Age (quadratic)	−0.00640	0.00269	−0.10	−2.382	0.017
Gender: male	−6.508	1.146	−0.15	−5.678	< 0.001
Displaced: yes	3.070	0.960	0.08	3.199	0.001
Education: college	−0.885	1.283	−0.02	−0.690	0.490
Education: bachelor	−1.564	1.465	−0.03	−1.068	0.286
Education: Master/PhD	−3.603	1.302	−0.07	−2.767	0.006
Marital: single	0.966	0.967	0.02	0.999	0.318
Family trauma: none	−1.833	1.090	−0.05	−1.681	0.093
Occupation: unemployed	1.789	1.345	0.04	1.330	0.184
Occupation: employed	2.511	1.065	0.06	2.358	0.019

*Note:* Unstandardised (*b*) and standardised coefficients (*β*) are reported. ‘Female’ is the reference level for Gender, ‘no’ for displacement, ‘never occupied’ for Occupation, ‘secondary’ for Education and ‘Holodomor’ for genocidal trauma. Adjusted *R*‐squared = 0.058. *N* = 1390.

### 3.2 Moral Injury

The backward elimination algorithm retained three predictors (out of 8) in the regression model fitted to the MISS scores: Age (linear and quadratic terms), Education and genocidal trauma. The second linear regression with only these three predictors was re‐fitted to the MISS scores, see Table 3 for regression coefficients.

**TABLE 3:** Coefficients of the regression model fitted to MISS scores.

Predictor	*b*	SE	*β*	*t*	*p*
Intercept	59.392	3.648	—	16.281	< 0.001
Age (linear)	−0.712	0.176	−0.20	−4.054	< 0.001
Age (quadratic)	0.00767	0.00215	0.14	3.559	< 0.001
Marital: single	−2.711	1.583	−0.05	−1.712	0.087
Family trauma: none	−4.751	1.235	−0.12	−3.848	< 0.001
Education: college	2.708	1.033	0.07	2.622	0.009
Education: bachelor	0.833	1.176	0.02	0.708	0.479
Education: Master/PhD	−0.145	1.046	0.00	−0.138	0.890
Single × Trauma (none)	3.970	1.745	0.06	2.275	0.023

*Note:* Unstandardised (*b*) and standardised coefficients (*β*) are reported. ‘No’ is the reference level for displacement, ‘never occupied’ for Occupation, ‘secondary’ for Education and ‘Holodomor’ for genocidal trauma. Adjusted *R*‐squared = 0.036. *N* = 1390.

**FIGURE 1:**
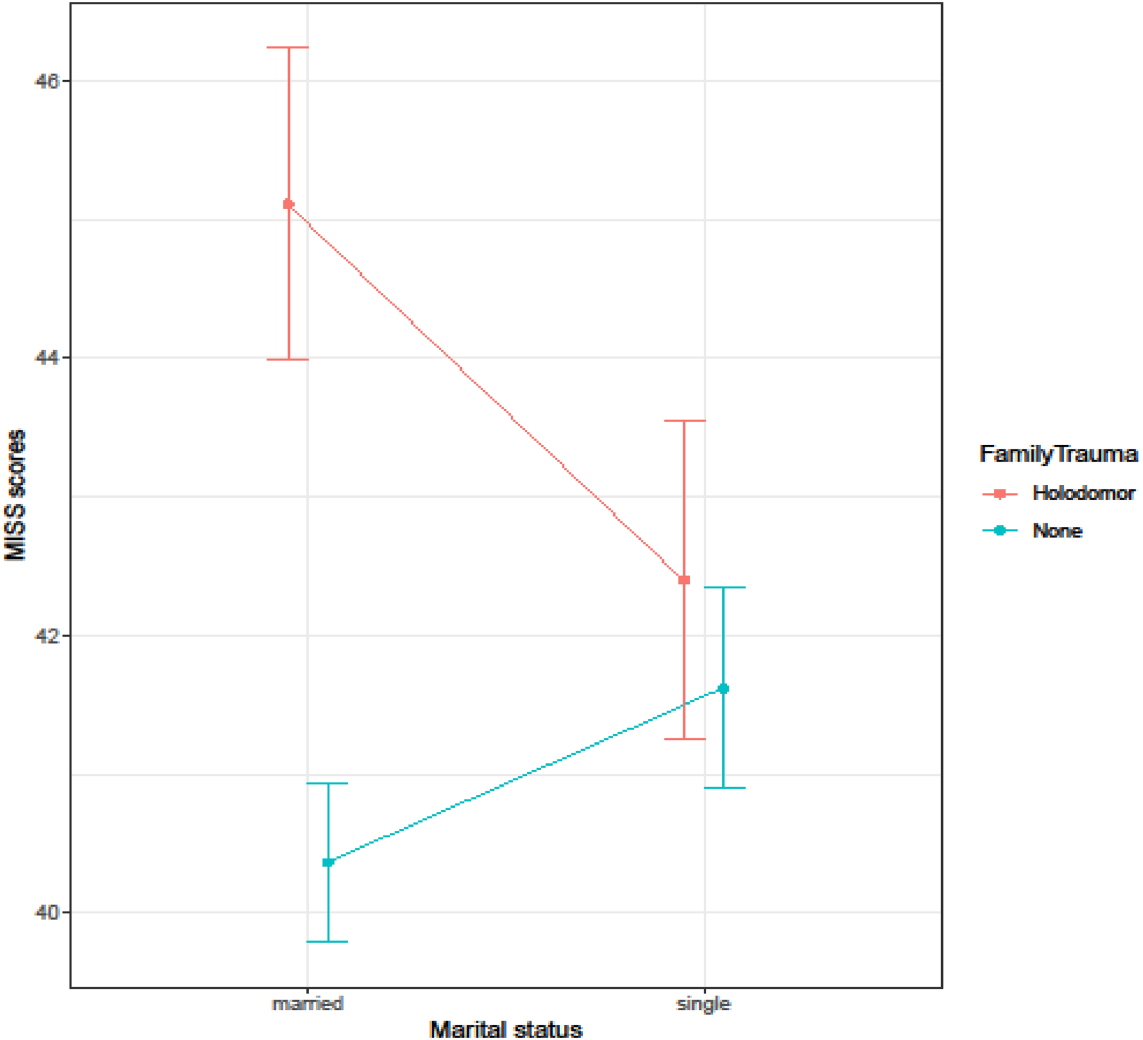
Partial interactive effects of the presence of genocidal trauma by marital status of the respondent. Error bars stand for ±1 SE.

The key finding was the interaction between the presence of genocidal trauma and one's family history and one's marital status (*b* = 3.970, SE = 1.745, *t* = −2.275, *p* = 0.023). Figure 1 visualises the interaction. For non‐married individuals, the presence or absence of trauma did not affect the score in the moral injury test. Conversely, for married respondents, the presence of the Holodomor trauma in family history came with a significantly higher score on the moral injury test, as indicated by the planned *t*‐test comparison (45.1 vs. 40.3 points, *p* < 0.001). The difference of roughly 4 points accounts for roughly 10% of the effective scale of the MISS scores.

Control predictors showed effects similar to the ones reported in Zasiekina, Kokun, et al. (2023) and Zasiekina, Zasiekin, and Kuperman (2023). Namely, moral injury scores decreased with age, and lower levels of education were associated with higher scores on the moral injury scale.

**FIGURE 2:**
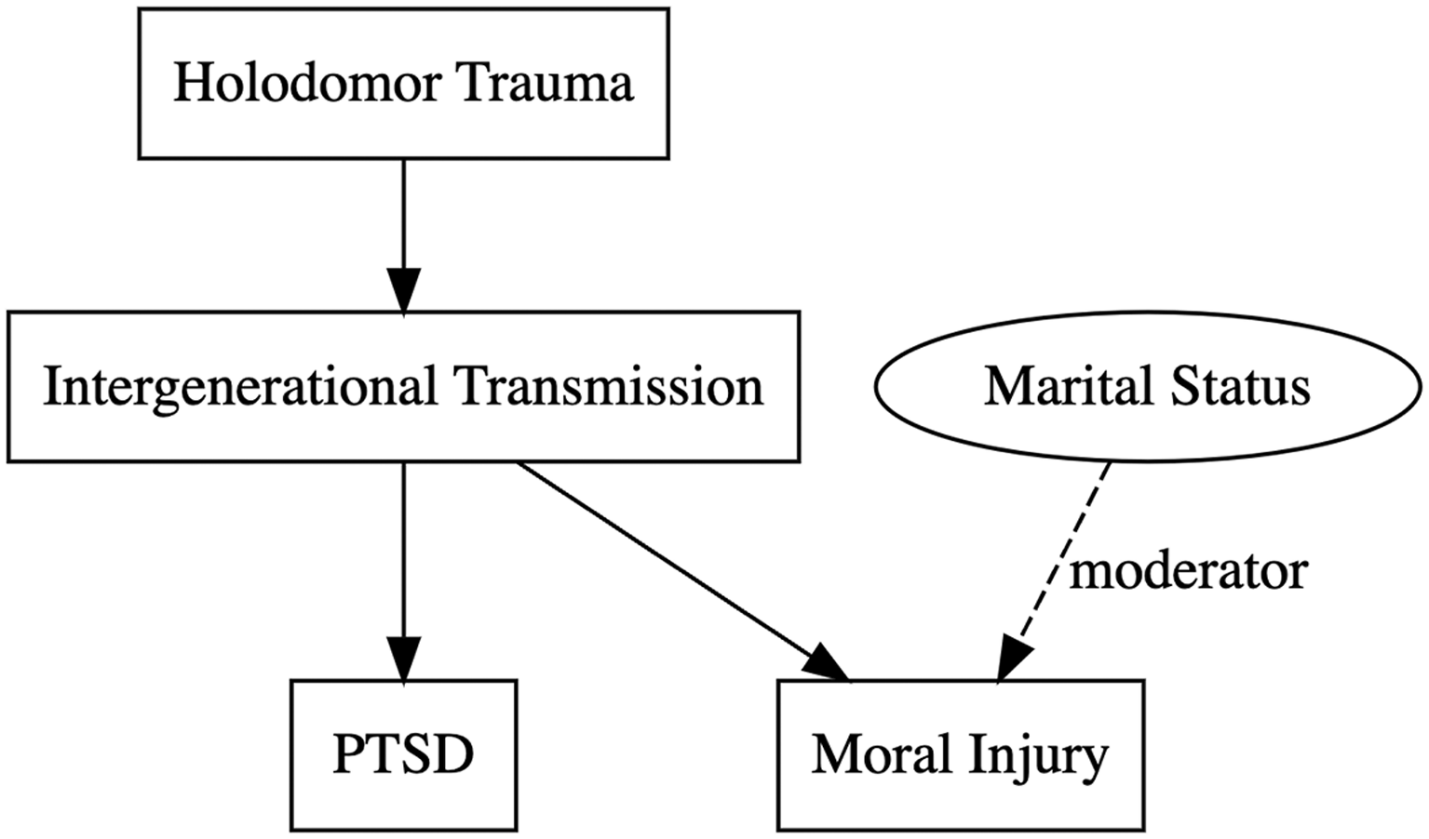
Interaction between Holodomor trauma and marital status on moral injury and PTSD.

This finding suggests that (i) the severity of moral injury and PTSD symptoms are related but conceptually independent psychological dimensions that are accompanied by different protective and risk factors, and (ii) there is an interaction of genocidal trauma and marital status on moral injury (with married individuals being particularly sensitive to the trauma in family history) but not for PTSD symptomatology (see Figure 2).

## 4 Discussion

The results of our study indicate that genocidal trauma has different effects on moral injury and PTSD manifestations in individuals exposed to war‐related trauma. The results from the current research indicate that the presence of genocidal trauma substantially contributes to moral injury symptoms in Ukrainian civilians while having no significant effect on PTSD. Moreover, there is a significant interaction effect between the genocidal trauma of the Holodomor and marital status on moral injury, indicating that married individuals with an ancestral genocidal trauma have higher moral injury scores compared to non‐married ones. This suggests that the compounded effects of previous genocidal trauma and current familial responsibilities may heighten the psychological burden, particularly in civilians exposed to war atrocities. These findings highlight the interplay between genocidal trauma and current psychosocial factors in shaping moral injury during times of war.

Our finding aligns with previous research highlighting the distinction between PTSD and moral injury. The question raised in Molendijk's (2022) paper regarding whether moral injury exemplifies concept creep finds evidence‐based support for its recognition as a distinct construct in our study. This corroborates recent findings in different approaches not only assessing but also treating moral injury and PTSD (Walker et al. 2024).

Danieli (2009) points out mistrust as a moral experience in the Holocaust survivor's offspring and underscores that a poor war‐related environment (‘third traumatic sequence’) could intensify the previous traumatic events, while a good environment might mitigate their poor outcomes. Nevertheless, recent studies overlook moral injury in the offspring of Holodomor survivors who are currently under the ‘third traumatic sequence’ of the full‐scale invasion of Ukraine. This study is crucial because it highlights the long‐term psychological impact of genocidal events—not only on those who directly experienced the trauma but also on their descendants, who may carry the emotional and moral burdens of that history.

To our knowledge, this is the first study to explore the effect of past genocidal trauma on moral injury in civilians, specifically among the third and fourth generations of Holodomor survivors. The different effects of genocidal trauma on PTSD and moral injury may stem from the different dimensions of these constructs. PTSD, assessed using the PCL‐5, focuses on symptoms like intrusive thoughts, hypervigilance and emotional changes, while moral injury, measured by the MISS, captures feelings of shame, guilt and betrayal.

Descendants of Holodomor survivors may experience heightened moral injury for two main reasons. First, many report inherited emotions such as anger, resentment and a sense of betrayal transmitted through family narratives about historical suffering. These emotions are often directed toward perceived sources of injustice associated with the historical events, reflecting an intergenerational moral appraisal of the trauma experienced by their ancestors. Second, the inherited burden of forced immoral behaviours, which were inevitable for survival, underscores the unique moral and emotional challenges associated with genocidal trauma. Recent findings suggest that these forced immoral acts during the genocide, such as stealing, killing, lynching and abandoning children to ensure the survival of oneself and loved ones (Kis 2021), may echo in contemporary conflicts, including the Russia‐Ukraine war, as individuals face similar extreme conditions and moral dilemmas.

Our findings are not in line with those of Shrira et al. (2024), who reported higher rates of PTSD in Holocaust survivors' offspring, particularly among those with combat exposure or civilians experiencing war‐related trauma during the initial 2 months of the Israel‐Hamas war. The participants in our sample were civilians assessed during the first 2 years of the Russia‐Ukraine war. The absence of higher PTSD rates in our sample, in contrast to the findings by Shrira et al. (2024), may be explained by differences in exposure duration, cultural context, or potentially the availability of coping mechanisms during prolonged conflict situations. Additionally, Shrira et al. (2024) explored the second and the third generations, while the participants in our study are the representatives of the third and fourth generations of the genocidal trauma. The discrepancy between the two studies highlights the complex interplay of temporal, contextual and individual factors in intergenerational trauma outcomes that need further exploration.

The absence of vulnerability to PTSD in civilian offspring of Holodomor victims, in contrast to the vulnerability demonstrated by active‐duty soldiers with the Holocaust ancestral trauma, as reported by Solomon (1988), could be explained by the nature of the trauma experienced. Civilians are often exposed to more passive, though no less destructive, forms of trauma, such as displacement, starvation and systemic oppression (Zasiekina, Kokun, et al. 2023; Zasiekina, Zasiekin, and Kuperman 2023). These experiences may influence psychological mechanisms more aligned with moral injury than PTSD.

Displacement may be considered a potential factor influencing intergenerational moral injury. While displacement, whether due to ongoing conflict or historical genocidal events like the Holodomor, represents a profound psychological stressor, our findings indicate that it significantly predicts PTSD symptoms in Ukrainian civilians but not moral injury. Displacement might involve not only coping with the trauma of forced relocation but also navigating morally challenging situations, such as leaving elderly relatives behind or exposing oneself and one's children to ongoing danger. These circumstances can evoke moral distress, a state arising from initial moral frustration in response to self‐relevant moral stressors, producing strong and persistent moral emotions that, under morally injurious conditions, may progress toward full moral injury (Litz and Kerig 2019). For many Ukrainians, repeated forced displacements, initially from the Donetsk and Luhansk oblasts in 2014 and later during the full‐scale invasion in 2022, may re‐activate ancestral moral injury linked to genocidal trauma, particularly when decisions involve no clear ‘right’ choice (Karstoft et al. 2023; Zasiekina, Kokun, et al. 2023; Zasiekina, Zasiekin, and Kuperman 2023). However, our results indicate that displacement itself predicts PTSD symptoms rather than moral injury, suggesting that the acute, survival‐focused stressors of relocation may drive trauma‐related psychopathology, whereas the chronic, meaning‐focused burden of protecting a family in the context of historical trauma represents the domain in which moral injury manifests.

Further research may consider the potential interaction effects between displacement, marital status and other factors, including age, prior traumatic experiences, the circumstances of displacement (planned vs. sudden, leaving vs. taking dependents), family income and social status on the development of moral injury.

An important finding of our study is that marital status interacts with genocidal trauma to influence moral injury. Family is a core value for Ukrainians, and married individuals may experience complex moral injury as they struggle with the dual responsibilities of protecting their loved ones. This moral injury may manifest through feelings of guilt or shame when individuals perceive themselves as unable to shield their family from harm or when their actions violate their moral beliefs, as well as through rage toward the perpetrators. Additionally, the inherited genocidal trauma of the Holodomor may instil a deep moral obligation in Ukrainians to address the emotional needs of family members who have endured atrocities. However, fulfilling this obligation is often impossible under the conditions of war, intensifying the burden of moral injury.

The findings of this research have important theoretical and practical implications. They highlight the significance of the family layer in the study of intergenerational moral injury. Furthermore, they open the possibility of tailoring family‐based interventions for civilians with intergenerational moral injury stemming from previous genocidal trauma and re‐activated by current war‐related events.

## 5 Limitations and Future Research

This study has several limitations, including the reliance on self‐report measures and the cross‐sectional design, which limits our ability to explore causality. Although the study provides an extensive discussion on the role of marital status in moral injury, the reverse relationship is also plausible, that is, moral injury could affect marital relationships. Therefore, the potential bidirectionality of this association needs further investigation. Additionally, future research should consider longitudinal studies to examine the trajectory of moral injury and the type of response to potentially morally injurious events in direct descendants of Holodomor survivors. Cross‐cultural studies could also expand our understanding of how moral injury manifests in different genocidal contexts and how survivors from different ethnic and cultural backgrounds cope with the trauma of mass violence.

Another limitation relates to the measurement of genocidal trauma. The use of a free‐form item (‘Is there any traumatic event in your family history?’) does not capture the degree of exposure, narrative engagement, or emotional connection to the Holodomor. Future studies could employ more nuanced tools, such as scales assessing intergenerational trauma awareness, the intensity of exposure, or the salience of familial narratives, to better quantify inherited trauma. Moreover, the Other category of Family Trauma variable contains 20 Holocaust mentions and many current war events. While the combination was necessary for statistical purposes, the resulting category is not homogeneous. A larger sample would allow for a more fine‐grained characterisation of similarities and differences between traumatic events like the Holodomor, Holocaust and the ongoing war with Russia.

Additional limitation concerns the moderate reliability of the MISS in this sample (ICC = 0.53). This level of internal consistency may introduce measurement error, potentially attenuating observed associations between moral injury and other variables. As a result, findings involving the MISS should be interpreted with caution. It is possible that the reliability of the MISS instrument in the current sample is somewhat undermined because it applies a scale developed for military personnel to civilians and also because the instrument is used in the context of active ongoing war.

## 6 Conclusion

The study highlights the intergenerational transfer of moral injury in Holodomor victims' offspring exposed to multiple exposures to war‐related trauma during the ongoing invasion of Ukraine. Our findings reveal a significant interaction between genocidal trauma and moral injury, particularly within the context of familial relationships. Notably, this interaction does not extend to PTSD, suggesting that the moral and ethical dimensions of trauma are transmitted across generations in a way that is deeply embedded in family dynamics. This underscores the uniquely ‘family‐centred’ nature of intergenerational moral injury, where the marital and familial context plays a leading role in shaping how such trauma is experienced and aggravated. These results call for further investigation into intergenerational moral injury and PTSD, as well as the need for targeted interventions that address both genocidal trauma and its current manifestations during the Russia‐Ukraine war, particularly within the family context.

## Author Contributions

**Larysa Zasiekina:** conceptualization, supervision, validation, project administration, methodology, writing – review and editing, funding acquisition, writing – original draft, investigation. **Serhii Zasiekin:** investigation, data curation, writing – review and editing. **Victor Kuperman:** supervision, resources, data curation, software, formal analysis, project administration, methodology, visualization, writing – review and editing, funding acquisition, writing – original draft, investigation.

## Funding

This work was supported by the British Academy, UK and The New Frontiers in Research Fund—Exploration NFRFE‐2‐22‐00802. This study is also a part of the project ‘The Impact of the Genocidal Trauma of the Holodomor on the Mental Health of Ukrainians: From Transgenerational Mechanisms to Community‐Oriented Interventions’ (2025–2027) funded by the Ministry of Education and Science of Ukraine, Reg. No. 0125U001724.

## Ethics Statement

All procedures performed in studies involving human participants were in accordance with the ethical standards of the institutional research ethics committee at Lesya Ukrainka Volyn National University and research ethics board at McMaster University and with the 1964 Helsinki Declaration and its later amendments or comparable ethical standards. Informed consent was obtained from all individual adult participants included in the study.

## Conflicts of Interest

The authors declare no conflicts of interest.

## Data Availability

The data that support the findings of this study are available from the corresponding author upon reasonable request.
